# The Impact of Lower Extremity Mechanical Axis Alignment on the Success of Platelet‐Rich Plasma Injections in Knee Osteoarthritis Patients

**DOI:** 10.1111/os.70185

**Published:** 2025-10-12

**Authors:** Alparslan Yurtbay, Furkan Erdoğan, Ferhat Say

**Affiliations:** ^1^ Department of Orthopaedics and Traumatology Samsun University Samsun Turkey; ^2^ Department of Orthopaedics and Traumatology Ondokuz Mayıs University Samsun Turkey

**Keywords:** angle, axis, knee, mechanical, osteoarthritis, platelet‐rich plasma

## Abstract

**Objectives:**

Knee osteoarthritis (OA) is a common cause of pain and disability, and conventional conservative treatments often provide only limited and temporary relief. Platelet‐rich plasma (PRP) injections have emerged as a promising biological therapy; however, patient response is highly variable, and biomechanical factors such as lower extremity malalignment may influence treatment outcomes. This study aimed to evaluate the effect of the lower extremity mechanical axis angle (MAA) on the clinical efficacy of PRP injection therapy in improving knee function and pain in patients with OA.

**Methods:**

A total of 210 patients with knee OA who consented to PRP treatment between January 1, 2018, and January 1, 2023, were enrolled. Patients were stratified into three groups according to baseline varus angle: Group 1, 0°–5° (*n* = 70); Group 2, 6°–10° (*n* = 70); and Group 3, 11°–15° (*n* = 70). Clinical evaluations were performed at baseline and at 1, 3, 6, 12, and 24 months post‐treatment using the Knee Injury and Osteoarthritis Outcome Score (KOOS), Kujala Patellofemoral Score, knee joint range of motion (ROM), MAA measurement, and a Visual Analogue Scale (VAS) for pain.

**Results:**

All groups demonstrated significant improvements in pain and functional scores over the 24‐month follow‐up compared to baseline (*p* < 0.001), with the most notable gains observed at 3 and 6 months. At 3, 6, and 12 months, Group 1 achieved significantly better VAS and KOOS Pain subscale scores than Group 3 (*p* < 0.05). Both Groups 1 and 2 had higher KOOS Total scores than Group 3 at these time points (*p* < 0.05). Spearman correlation analysis revealed moderate negative associations between baseline MAA and changes from baseline to 6 months in VAS (*ρ* = −0.58), KOOS Total (*ρ* = −0.54), and Kujala scores (*ρ* = −0.53) (all *p* < 0.001). Statistical analyses were conducted using ANOVA or Kruskal–Wallis tests as appropriate, and effect sizes (Cohen's d) with 95% confidence intervals were calculated.

**Conclusion:**

PRP injection therapy yields significant improvements in pain and functional outcomes in patients with knee OA. However, increased MAA is associated with reduced clinical benefit, indicating that baseline lower extremity alignment should be considered in treatment planning.

## Graphical abstract

This illustration summarizes the study design and findings on knee osteoarthritis patients with varying varus alignment receiving PRP injections. Significant clinical improvements (VAS ↓, KOOS ↑) were seen in those with mild alignment deviations, while patients with severe varus deformities showed limited benefits. The graphical abstract was produced using an AI‐based illustration tool and was subsequently reviewed and refined by the authors.

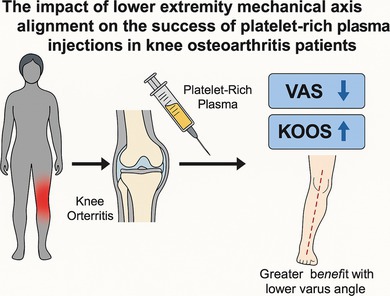

## 1 Introduction

Degenerative cartilage lesions can lead to the degradation of the articular surface and subchondral bone, ultimately resulting in osteoarthritis (OA) [1]. This prevalent condition affects over 10% of people aged 60 and older and is a leading cause of disability, significantly impacting quality of life and healthcare costs [2]. Joint damage associated with OA should be considered as an organ failure, like kidney or heart failure. The pathogenesis of OA is multifactorial, and its underlying processes remain poorly understood. While it is often associated with aging and mechanical stress, various systemic and local factors also contribute to its onset and progression [3]. Understanding the etiology and pathophysiology of OA is of utmost importance for developing effective prevention and treatment strategies. Irrespective of their cause, various processes can lead to symptomatic and advanced OA. Although early diagnosis of knee OA is possible, the available conservative treatments, such as physiotherapy, anti‐inflammatory drugs, and painkillers, have limited and temporary efficacy. Furthermore, they cannot prevent the progression of the disease [1]. For this reason, the demand for knee replacement is high and continuously growing. However, whereas the surgical approach can provide a high success rate and satisfaction for older patients, high functional requests and longer life expectancy of young patients are issues for joint arthroplasty. The need for knee replacement procedures is increasing for these reasons. Nevertheless, surgical intervention is most appropriate for older patients, who typically experience tremendous success and satisfaction. Conversely, younger patients with higher expectations for joint function and a longer lifespan present a formidable challenge for joint arthroplasty.

Biological treatments have recently emerged as promising for articular degenerative defects and early knee OA. These methods aim to reduce symptoms, restore knee function, and potentially prevent OA progression, thereby delaying the need for metal resurfacing. Various intra‐articular products are used in clinical practice, including corticosteroids, viscosupplementation, and new orthobiologic solutions like platelet concentrates. One of these treatment methods is platelet‐rich plasma (PRP); It has received increasing attention due to the high concentrations of cytokines [4] and growth factors [5] stored in platelet alpha granules, which have been shown to play a role in the homeostasis of articular cartilage [6], involved in both the healing process and immune regulation. Growth factors revealed by the degranulation of platelets play an important role in the angiogenesis and proliferation of chondrogenic cells, secretion of the cartilaginous matrix, tissue remodeling, and wound healing [7]. Besides the biological rationale, the current preclinical evidence further supports the role of PRP in mitigating inflammation in patients with degenerative joint diseases [8]. It is crucial to emphasize that the primary effect of PRP intra‐articular treatment may not directly stimulate tissue regeneration, as suggested by the available evidence. However, it is possible that the treatment exerts its impact through various bioactive molecules that help maintain tissue balance and slow down inflammation, catabolism, and degeneration, leading to the alleviation of symptoms and functional improvement.

There is a significant relationship between the frontal alignment of the lower extremity and the pathogenesis of OA [9, 10]. The relationship between lower extremity malalignment and the development of OA and the progression of the process has been shown in many studies [9, 10, 11, 12, 13, 14]. At present, there is a lack of clinical research examining the potential correlation between PRP treatment and lower extremity mechanical axis angle. PRP treatment affects the biochemical environment within the joint but cannot directly affect the structure of the joint.

The primary purpose of this study was to understand the relationship between the lower limb mechanical axis angle (MAA) and the effectiveness of PRP treatment in OA patients. Given the biomechanical role of malalignment in altering joint load distribution, we hypothesized that greater varus alignment, reflected by a higher mechanical axis angle (MAA), would be associated with smaller improvements in pain, function, and range of motion after PRP treatment. By identifying alignment‐related differences in treatment response, this study seeks to contribute to more precise patient selection criteria and optimize clinical decision‐making for PRP therapy in knee osteoarthritis. PRP preparation and application techniques have been standardized to provide a high level of scientific evidence.

## 2 Patients and Methods

### 2.1 Study Design

The study was designed retrospectively, based on the data set in which the information of the patients who received three doses of PRP treatment in the osteoarthritis patient group was recorded. Among the patients who applied to our clinic between 01.01.2018 and 01.01.2023, patients who volunteered to participate in PRP treatment and whose informed consent was obtained were included in the study. Ethical approval for this study was obtained from the Samsun University clinical research ethics committee (approval number: SÜKAEK‐2023 14/16, date: 09.08.2023). All the details of this clinical study were explained to the patients, and all participants in the study voluntarily provided written informed consent. All the study procedures complied with the principles of the Declaration of Helsinki.

### 2.2 Sample and Sampling

The study analyzed 210 patients who sought treatment at the Department of Orthopedics and Traumatology at Samsun University Faculty of Medicine between January 1st, 2018, and January 1st, 2023. The inclusion criteria were as follows: (1) diagnosis of knee osteoarthritis (OA) according to the American College of Rheumatology classification criteria [15]; (2) radiographic evidence of symptomatic OA at Kellgren–Lawrence (K/L) stages 1–4 [16]; (3) age between 18 and 80 years; (4) an average Visual Analogue Scale (VAS) pain score exceeding 4 out of 10 (representing the worst possible pain) over a seven‐day period within the previous month. It's worth noting that none of the patients received bilateral injections.

The study has certain exclusion criteria that patients must meet. These include osteoarthritis caused by joint inflammatory diseases, metabolic bone disease, coexisting back pain, hematological disease (coagulopathy), bilateral symptomatic lesions, intra‐articular injection within the past 3 months, arthroscopic lavage within the past year, use of immunosuppressive drugs, current use of anticoagulant medications or non‐steroidal anti‐inflammatory drugs (NSAIDs) in the 5 days before blood sampling, major axis deviation of the knee (> 15° varus or > 5° valgus deviation), hemoglobin level < 11.5 g/dL and platelet level < 100,000/μL, comorbidities associated with the above factors, infection, tumor, crystal arthropathies, anemia, intense joint effusion, or known or possible pregnancy. In order to ensure the accuracy and validity of the study, patients with body mass index (BMI) that fall outside the range of 18.5–30 were not included.

The study's power analysis was based on the sample sizes used in previous studies. To achieve a standard deviation of 6 units and a difference of *d* = 3 units between the two means, a sample size of 210 was required for a 95% power in a 95% confidence interval. All patients were selected based on predetermined inclusion and exclusion criteria.

The patients were divided into three groups according to lower limb mechanical axis angle measurements: Group 1, 0–5 degrees varus (*n* = 70); Group 2, 6–10 degrees varus (*n* = 70); and Group 3, 11–15 degrees varus (*n* = 70).

### 2.3 PRP Preparation and Implementation Procedure

**FIGURE 1:**
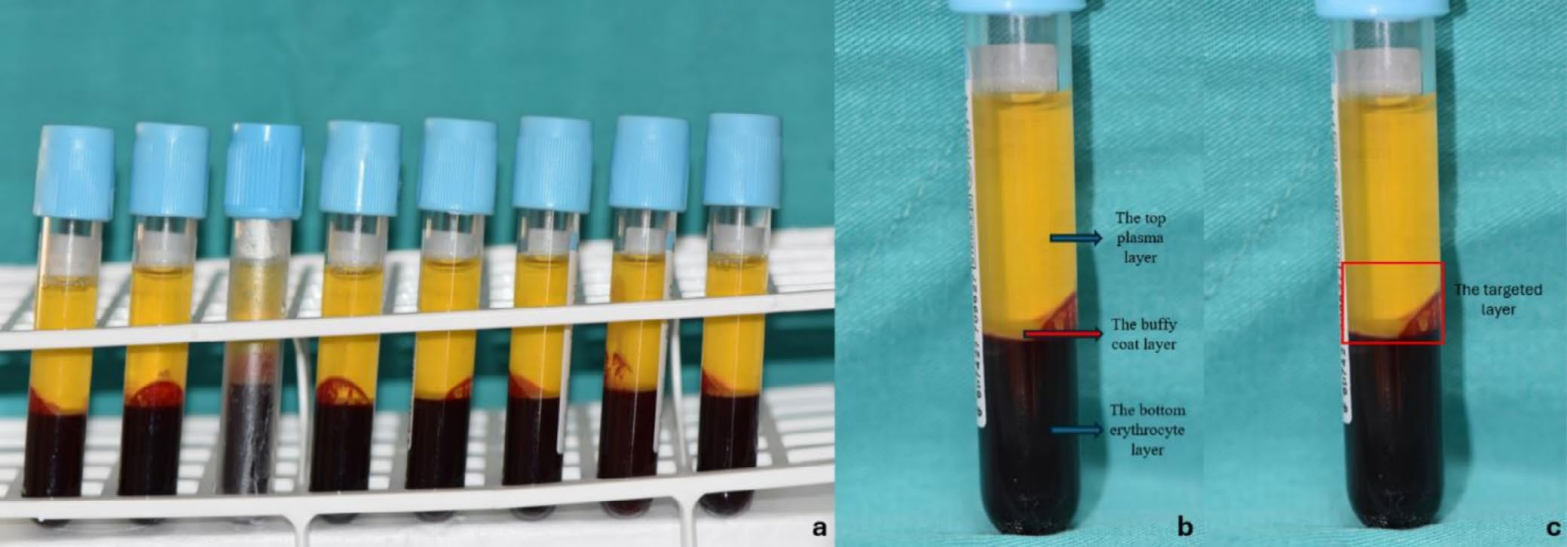
(a) Eight tubes of a total of 32 mL blood prepared for centrifugation. (b) The appearance and layers formed after centrifugation. The red arrow indicates the buffy coat layer (0.2 mL). (c) After centrifugation, 0.2 mL of the middle layer and 0.8 mL of the platelet‐rich upper layer were collected from each patient to standardize the separation process.

Thirty‐two mililiter of peripheral venous blood was collected from the antecubital vein. During the blood collection, we adhered to strict aseptic protocols to ensure safety, taking great care to minimize trauma to protect the integrity of the platelets. The blood was placed in eight sterile tubes, each containing 4.5 mL of 3.2% sodium citrate as an anticoagulant (Figure 1a). Following collection, the tubes were centrifuged for 10 min at 1800 rpm.

After centrifugation, whole blood is segregated into three distinct layers based on gravity: layer 1 is plasma (the top layer), layer 2 consists of platelets and leukocytes (the middle layer, commonly referred to as the “buffy coat”), and layer 3 comprises erythrocytes (the bottom layer) (Figure 1b). Following the centrifugation process, a total of approximately 4 mL of blood was obtained, with about 2 mL of plasma at the top, 0.2 mL of buffy coat in the middle, and 1.8 mL of erythrocytes at the bottom. These values are approximate, as they can vary among patients due to differences in erythrocyte volume. This technique employs a modified version of the Anitua method, which is frequently favored for the separation process [17]. To standardize the separation process, 0.2 mL of the middle layer from each patient was collected, along with the 0.8 mL top layer rich in platelets (Figure 1c). This method aims to obtain consistent layers across patients, although variations may still exist between individuals. An additional 1.2 mL of top plasma and 1.8 mL of the erythrocyte layer were discarded. The remaining 0.8 mL of plasma and 0.2 mL of buffy coat were then transferred to a separate sterile tube. In total, 8 mL of PRP was collected, with 1 mL taken from each tube. This procedure was consistently carried out by the same study nurse for every patient. From the final 8 mL of PRP obtained, 5 mL was injected into the patient's knee through the anterolateral portal under sterile conditions, while the remaining 3 mL was sent to the laboratory for analysis of platelet and leukocyte counts.

For platelet activation, 50 μL of 5.5% calcium chloride (CaCl_2_) was added to 1 mL of PRP. The entire separation procedure was conducted within a biosafety cabinet. The resulting PRP exhibited a high leukocyte concentration, with an average of 9500–12,000 leukocytes/μL, thereby classifying it as leukocyte‐rich PRP. The platelet counts averaged 145 × 10^5^/μL, exceeding five times the patients' baseline levels. As per the Mishra classification, this product is categorized as type 2A [18]. Following the injection, patients were observed for an average of 30 min to ensure that no side effects occurred.

The PRP was administered into the knee joint using an anterolateral approach. Multiple injections of PRP were performed, with three doses given at one‐month intervals. During the follow‐up period, particularly in the first 6 months, the use of NSAIDs was prohibited. Patients were prescribed paracetamol at a dosage of 500 mg three times daily for any discomfort. Additionally, all patients were advised to avoid using anti‐inflammatory or analgesic medications as much as possible.

### 2.4 Outcome Measures

The patient's pain complaints were evaluated using the Visual Analogue Scale (VAS) and the pain subscale of the Knee Injury and Osteoarthritis Outcome Score (KOOS). A satisfaction questionnaire was administered to the patients, highlighting a significant gap in clinical research regarding the potential relationship between PRP treatment and the mechanical axis angle of the lower extremities. In addition to pain, other symptoms were assessed, along with activities of daily living (ADL), sports and recreational functions (Sport/Rec), and knee‐related quality of life (QOL), all measured through the five subscales of the KOOS scoring system. The Kujala Patellofemoral Score (KUJALA) was utilized to evaluate the impact of PRP on patellofemoral joint function.

Each patient underwent long‐leg standing radiographs to measure the mechanical axis angle (MAA) of the lower limbs. The range of motion (ROM) at the knee joint was assessed using a goniometer during each outpatient follow‐up examination. All parameters were recorded prior to injection and at 1, 3‐, 6‐, 12‐, and 24 months post‐injection by the same observer. Any potential side effects resulting from the treatment were documented during these follow‐up examinations.

### 2.5 Lower Extremity Mechanical Axis Measurement

**FIGURE 2:**
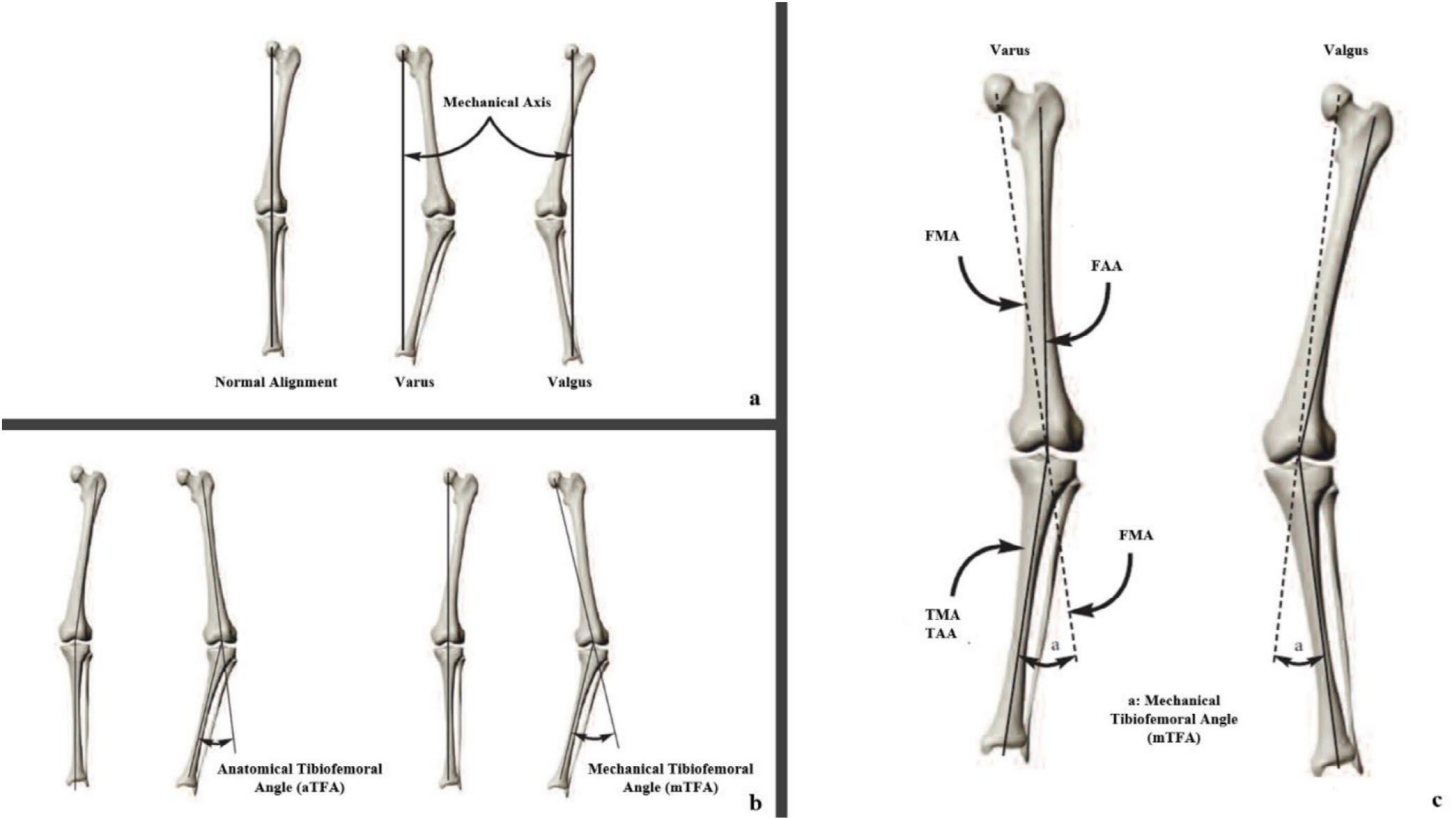
(a) Normal lower extremity alignment, varus and valgus lower extremity alignment according to the mechanical axis. (b) Anatomical axis angle and mechanical axis angle. (c) Measurement of mechanical axis angle. FAA: femoral anatomical axis; FMA: femoral mechanical axis; TAA: tibial anatomical axis; TMA: tibial mechanical axis.

The line extending from the midpoint of the femoral head to the midpoint of the ankle joint typically passes through the midpoint of the knee joint and represents the mechanical axis of the lower limb. Significant deviations may occur at the knee joint when this axis is used as a reference. Clinically, these angular deviations manifest as genu valgum (knock‐knees) or genu varum (bowlegs) (Figure 2a).

The mechanical tibiofemoral angle (mTFA) is defined as the angle formed between the femoral mechanical axis (FMA) and the tibial mechanical axis (TMA) (Figure 2b). This angle is also known as the hip‐knee‐ankle (HKA) angle. A normal mTFA falls within the range of 2 degrees of varus (inward angle) to 2 degrees of valgus (outward angle). Alterations in the geometry of the femur and tibia, along with the hip, knee, and ankle joints, can influence the anatomical tibiofemoral angle (aTFA), measured to assess alignment. As a result, mTFA measurements provide a more reliable and accurate evaluation of alignment in patients with OA [19].

In this study, when calculating the patients' deformity angles, the angle between FMA and TMA, namely mTFA, was used as the reference deformity angle. Also known as the “HKA” angle, it was used (Figure 2c).

### 2.6 Statistical Analysis

Data were analyzed using IBM SPSS Statistics, Version 23 (IBM Corp., Armonk, NY, USA). The normality of distribution was assessed with the Kolmogorov–Smirnov test. For categorical variables, between‐group comparisons were performed using the Pearson chi‐square test or Fisher's exact test with Monte Carlo correction, and pairwise comparisons were conducted using the Bonferroni‐corrected *Z* test. Normally distributed continuous variables were compared among groups using one‐way analysis of variance (ANOVA) followed by Tukey's HSD test for pairwise comparisons. For non‐normally distributed variables, the Kruskal–Wallis test was applied, with pairwise comparisons performed using the Dunn test. Correlations between non‐normally distributed variables were evaluated using Spearman's rho correlation coefficient.

In addition, effect sizes (Cohen's d) and their 95% confidence intervals were calculated for between‐group comparisons of changes from baseline to 6 months in VAS, KOOS Total, and KUJALA scores. Results are presented as frequency (percentage) for categorical variables and as mean ± standard deviation or median (minimum–maximum) for continuous variables. A *p* value < 0.050 was considered statistically significant.

## 3 Results

### 3.1 Patient Characteristics

The follow‐up of 210 patients was completed, and statistical analysis was performed. Table 1 demonstrates the effect sizes (Cohen's d) with 95% confidence intervals for between‐group comparisons from baseline to 6 months. Moderate‐to‐large negative effect sizes were observed for KOOS Total in patients with higher varus alignment (Group 3) compared to Groups 1 and 2, indicating reduced functional gains. VAS improvements were also smaller in Group 3, with moderate effect sizes, while KUJALA differences were smaller, suggesting less impact on patellofemoral function compared to overall knee function.

**TABLE 1:** Effect sizes (Cohen's d) and 95% confidence intervals for between‐group comparisons of changes from baseline to 6 months in VAS, KOOS Total, and KUJALA scores.

Measure	Group comparison	Cohen's d	95% CI Lower	95% CI Upper
VAS (0–6 months change)	1 vs. 3	0.319	−0.018	0.655
VAS (0–6 months change)	2 vs. 3	0.501	0.162	0.841
VAS (0–6 months change)	1 vs. 2	−0.194	−0.529	0.141
KOOS Total (0–6 months change)	1 vs. 3	−0.710	−1.055	−0.366
KOOS Total (0–6 months change)	2 vs. 3	−0.881	−1.232	−0.531
KOOS Total (0–6 months change)	1 vs. 2	0.265	−0.071	0.600
KUJALA (0–6 months change)	1 vs. 3	−0.237	−0.573	0.098
KUJALA (0–6 months change)	2 vs. 3	−0.349	−0.686	−0.012
KUJALA (0–6 months change)	1 vs. 2	0.119	−0.215	0.454

*Note*: Positive values indicate greater improvement in the first‐mentioned group, while negative values indicate greater improvement in the second‐mentioned group.

Baseline categorical characteristics differed significantly between the groups in terms of sex distribution, affected side, and Kellgren–Lawrence (K/L) grade (Table 2). The proportion of female patients was highest in Group 3 (87.1%) compared to Group 1 (68.6%) and Group 2 (65.7%) (*p* = 0.008). The side affected also varied significantly, with a higher proportion of left knees in the Group 2 (60%) and Group 3 (55.7%) groups compared to the Group 1 (*p* = 0.048). Regarding radiographic severity, patients in Group 3 had a higher prevalence of K/L grade 4 (18.5%) and grade 3 (28.5%), whereas Group 2 showed the lowest proportion of severe OA. There was no statistically significant difference between groups in BMI classification (*p* = 0.245), with the majority of patients in all groups classified as overweight.

**TABLE 2:** Comparison of categorical variables by groups.

	Groups			Test statistics	*p*
	Group 1	Group 2	Group 3		
	0–5 degree varus	6–10 degree varus	11–15 degree varus		
Sex					
Female	48 (68.6)^a^	46 (65.7)^a^	61 (87.1)^b^	9.804	0.008*
Male	22 (31.4)	24 (34.3)	9 (12.9)		
Side					
Right	42 (60)	28 (40)	31 (44.3)	6.219	0.048*
Left	28 (40)	42 (60)	39 (55.7)		
K/L grade					
Grade 1	7 (10)	5 (7.1)	2 (3)	17.465	0.001**
Grade 2	49 (70)^ab^	57 (81.4)^b^	35 (50)^a^		
Grade 3	14 (20)^ab^	6 (8.6)^b^	20 (28.5)^a^		
Grade 4	—^ab^	2 (2.9)^b^	13 (18.5)^a^		
BMI classification					
Normal	13 (18.6)	20 (28.6)	12 (17.1)	3.224	0.245*
Overweight	57 (81.4)	50 (71.4)	58 (82.9)		

*Note*: *Pearson Ki‐square test; ** Monte Carlo correction Fisher Exact test; a, b: There is no difference between groups with the same letter. Bold values indicate statistically significant results (*p* < 0.05).

Quantitative baseline characteristics are presented in Table 3. The mean follow‐up time differed significantly between groups (*p* = 0.030), with Group 1 having the longest mean follow‐up (21.43 ± 3.76 months) compared to Group 2 (19.8 ± 3.87 months). There was no significant difference in age (*p* = 0.079) or BMI (*p* = 0.526) across the groups. As expected, mechanical axis angle values were significantly different among all three groups (*p* < 0.001), increasing in accordance with the grouping criteria: 3.96° ± 1.12° in Group 1, 7.34° ± 1.5° in Group 2, and 11.7° ± 1.37° in Group 3 (Table 4).

**TABLE 3:** Comparison of quantitative variables by groups.

	Groups			Test statistics	p*
	Group 1	Group 2	Group 3		
	0–5 degree varus	6–10 degree varus	11–15 degree varus		
Follow‐up time	21.43 ± 3.76	19.8 ± 3.87	20.49 ± 4.27	7.033	**0.030**
	24 (12–24)^b^	18 (12–24)^a^	24 (12–24)^ab^		
Age	53.39 ± 12.37	53.91 ± 11.46	57.67 ± 8.89	5.086	0.079
	55 (20–80)	55 (24–79)	59 (39–78)		
BMI	27.49 ± 2.58	27 ± 2.69	27.57 ± 2.25	1.286	0.526
	28.42 (18.52–29.97)	27.78 (18.73–29.93)	28.11 (21.56–29.89)		
Mechanical axle angle	3.96 ± 1.12	7.34 ± 1.5	11.7 ± 1.37	179.677	**< 0.001**
	4 (1–5)^c^	7 (6–13)^b^	11 (5–15)^a^		

*Note*: *Kruskal Wallis test; Mean ± standard deviation; Median (minimum − maximum); a, b: There is no difference between groups with the same letter. Bold values indicate statistically significant results (*p* < 0.05).

**TABLE 4:** Baseline characteristics of the three groups.

	Groups			Test statistics	p*
	Group 1	Group 2	Group 3		
	0–5 degree varus	6–10 degree varus	11–15 degree varus		
Joint range of motion	120.71 ± 9.49	123.5 ± 10.88	115 ± 10.8	21.718	**< 0.001**
	120 (85–135)^a^	125 (95–135)^a^	115 (90–135)^b^		
VAS score	6 ± 1.73	6.71 ± 1.76	7.09 ± 1.42	1.914	0.384
	6 (3–10)	7 (3–10)	7 (4–10)		
KOOS score					
Symptom	63.62 ± 15.45	67.9 ± 16.63	60 ± 17.34	8.477	**0.014**
	62.5 (32.14–100)^ab^	67.86 (14.29–96.43)^a^	57.14 (25–92.86)^b^		
Pain	48.49 ± 16.27	51.15 ± 16.85	42.54 ± 13.86	9.800	**0.007**
	47.22 (22.22–86.11)^ab^	47.22 (8.33–91.67)^b^	44.44 (16.67–75)^a^		
ADL	50.21 ± 17.11	52.79 ± 19.29	44.52 ± 14.14	8.828	**0.012**
	45.59 (25–89.71)^ab^	51.47 (0–94.12)^a^	42.65 (22.06–77.94)^b^		
Sport/Rec	32.86 ± 21.31	35.14 ± 19.32	27 ± 16.78	6.315	**0.043**
	25 (0–80)^ab^	35 (0–75)^a^	25 (0–75)^b^		
QOL	35.18 ± 17.78	35.54 ± 18.07	30.27 ± 14.3	3.664	0.160
	31.25 (0–75)	37.5 (0–75)	25 (0–68.75)		
Kujala Score	55.97 ± 15.33	57.64 ± 13.78	53.11 ± 14.75	3.740	0.154*
	53 (21–87)	57.5 (21–82)	52 (20–80)		

*Note*: *Kruskal Wallis test; Mean ± standard deviation; Median (minimum − maximum); a, b: There is no difference between groups with the same letter. Bold values indicate statistically significant results (*p* < 0.05).

### 3.2 Pain and Functional Outcomes

**FIGURE 3:**
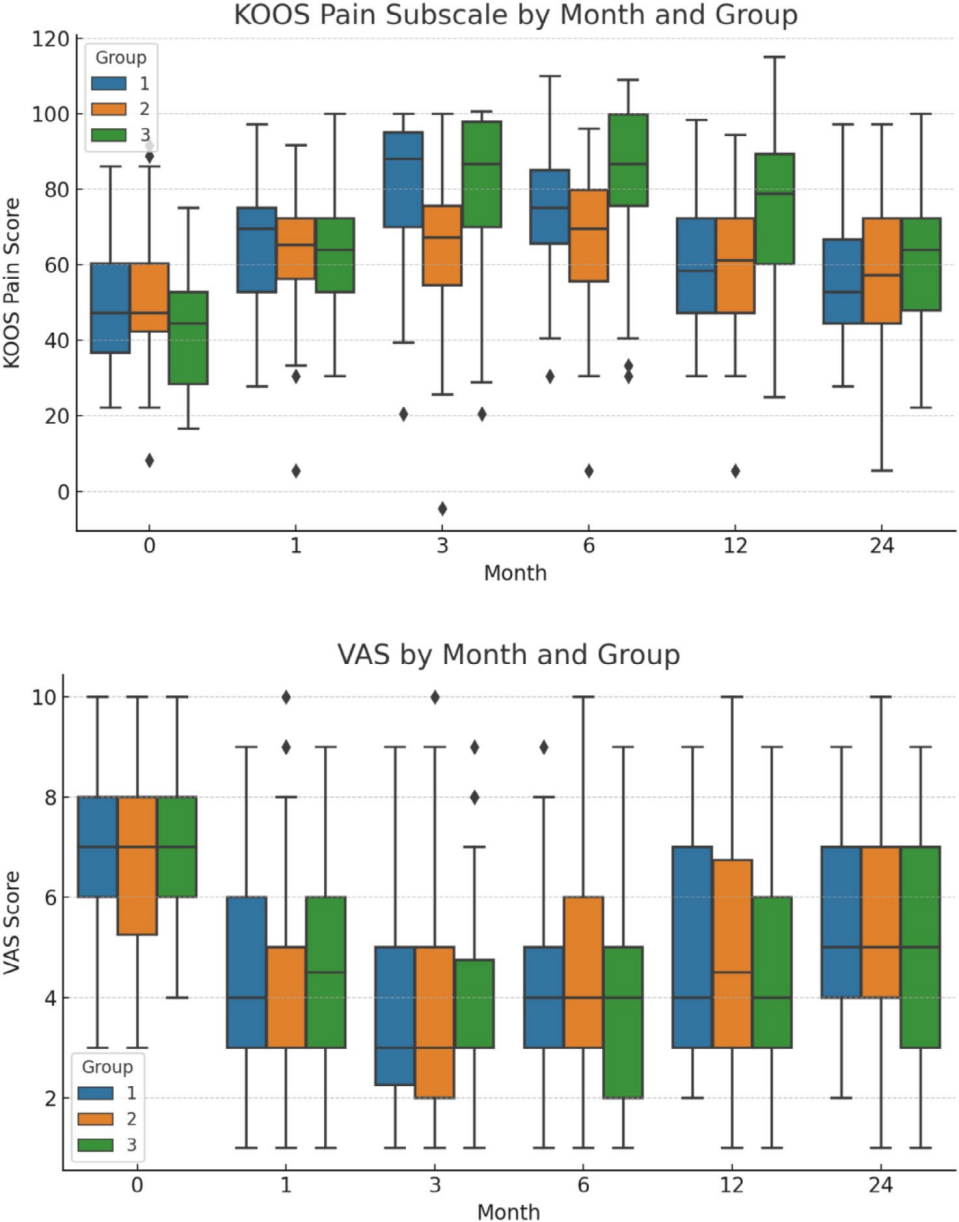
Box plots showing the distribution of KOOS Pain subscale scores and VAS scores across follow‐up months (0, 1, 3, 6, 12, and 24) for each mechanical axis alignment group. Boxes represent the interquartile range (IQR) with the horizontal line indicating the median, whiskers extend to 1.5 × IQR, and outliers are shown as individual points. Significant differences between groups at specific time points are indicated.

KOOS Pain and VAS scores improved significantly in all groups during follow‐up, with the most pronounced improvements observed at 3 and 6 months (Figure 3). At these time points, Group 1 had significantly better KOOS Pain and VAS scores compared to Group 3 (*p* < 0.05). Group 2 also tended to score higher than Group 3, although differences were smaller. By 12 months, the advantage of Groups 1 and 2 over Group 3 persisted but was reduced, and by 24 months, between‐group differences were no longer statistically significant.

**FIGURE 4:**
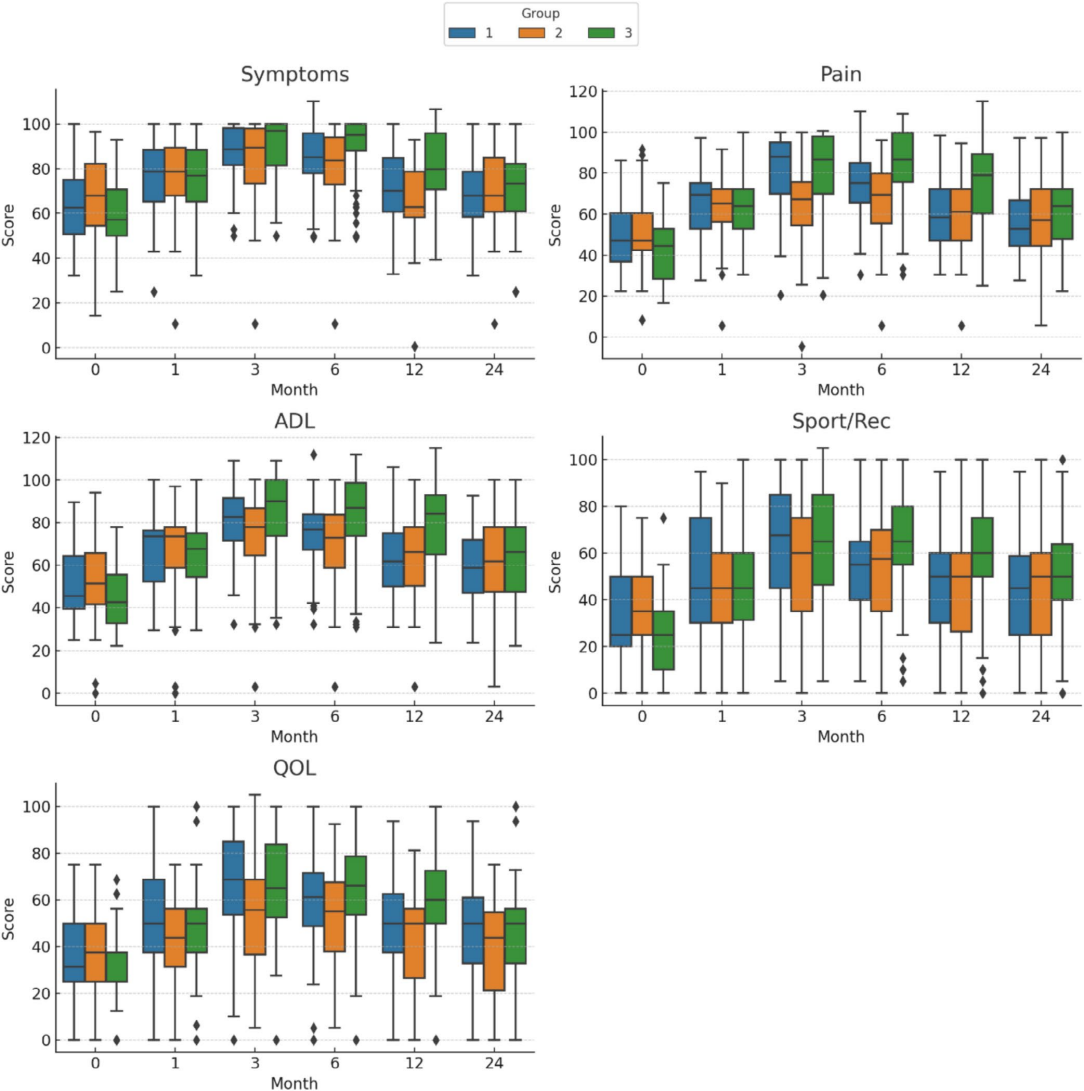
Box plots illustrating the distribution of KOOS subscale scores (Pain, Symptoms, Activities of Daily Living [ADL], Sport and Recreation [Sport/Rec], and Quality of Life [QOL]) at baseline and follow‐up months (1, 3, 6, 12, and 24) for each mechanical axis alignment group. Boxes represent the interquartile range (IQR) with medians shown as horizontal lines; whiskers extend to 1.5 × IQR and outliers are plotted as individual points. Significant differences between groups at each time point are indicated.

All KOOS subscale scores improved significantly from baseline in all groups during the follow‐up period (Figure 4). At 3 and 6 months, Groups 1 and 2 had significantly higher scores than Group 3 across most subscales (*p* < 0.05), indicating better pain control, symptom reduction, daily living function, sport/recreation ability, and quality of life. The advantage of Groups 1 and 2 over Group 3 persisted for several subscales at 12 months, particularly in Pain and ADL, but diminished thereafter. By 24 months, differences between groups were no longer statistically significant for any subscale.

### 3.3 Kujala Score Outcomes

The Kujala Score values of all patients were noted prior to injection and at 1, 3, 6, 12, and 24 months. There were significant differences between pre‐treatment and post‐treatment results in all groups during the 24‐month follow‐up period (*p* < 0.001), and better Kujala Score values were recorded at 3 and 6 months in all three groups. Compared to Group 3 at 3 and 6 months, Groups 1 and 2 had higher Kujala Score values (*p* < 0.05). When the Kujala Score values of the groups were compared, no statistically significant difference was determined between the groups after 24 months.

### 3.4 Correlation Analyses

**FIGURE 5:**
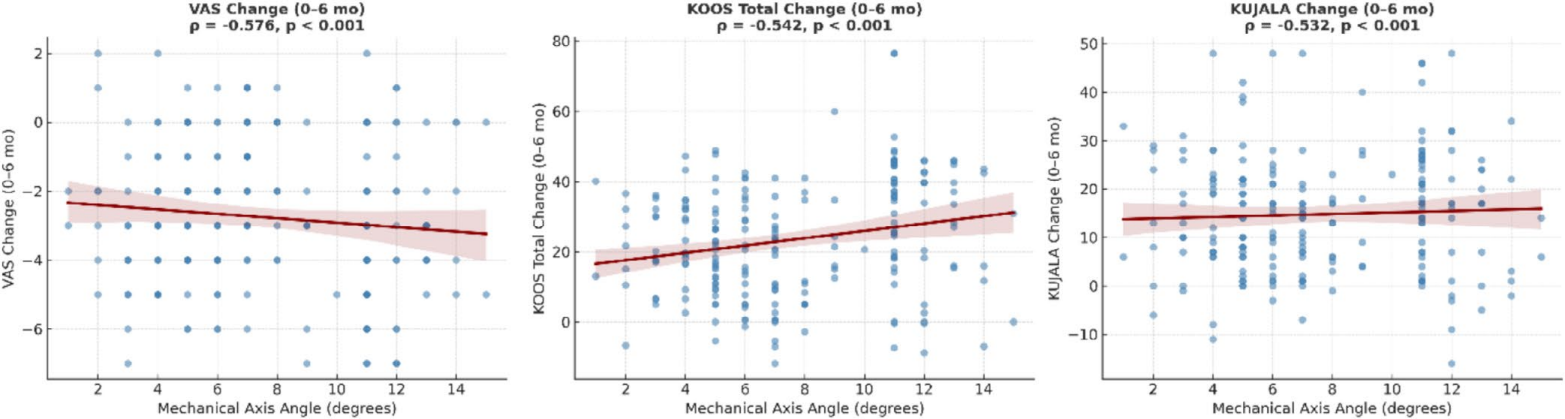
Scatter plots illustrating the relationship between Mechanical Axis Angle (MAA) and changes from baseline to 6 months in VAS, KOOS Total, and KUJALA scores. Each plot includes a fitted regression line with 95% confidence bands. Spearman correlation coefficients (*ρ*) and *p* values indicate statistically significant moderate negative correlations for all three measures (*p* < 0.001).

There were moderate, statistically significant negative correlations between baseline mechanical axis angle (MAA) and changes from baseline to 6 months in VAS (*ρ* = −0.576, *p* < 0.001), KOOS Total (*ρ* = −0.542, *p* < 0.001), and KUJALA scores (*ρ* = −0.532, *p* < 0.001) (Figure 5). These findings indicate that greater varus alignment is associated with smaller improvements in pain, overall knee function, and patellofemoral function following PRP treatment.

### 3.5 Range of Motion (ROM)

**FIGURE 6:**
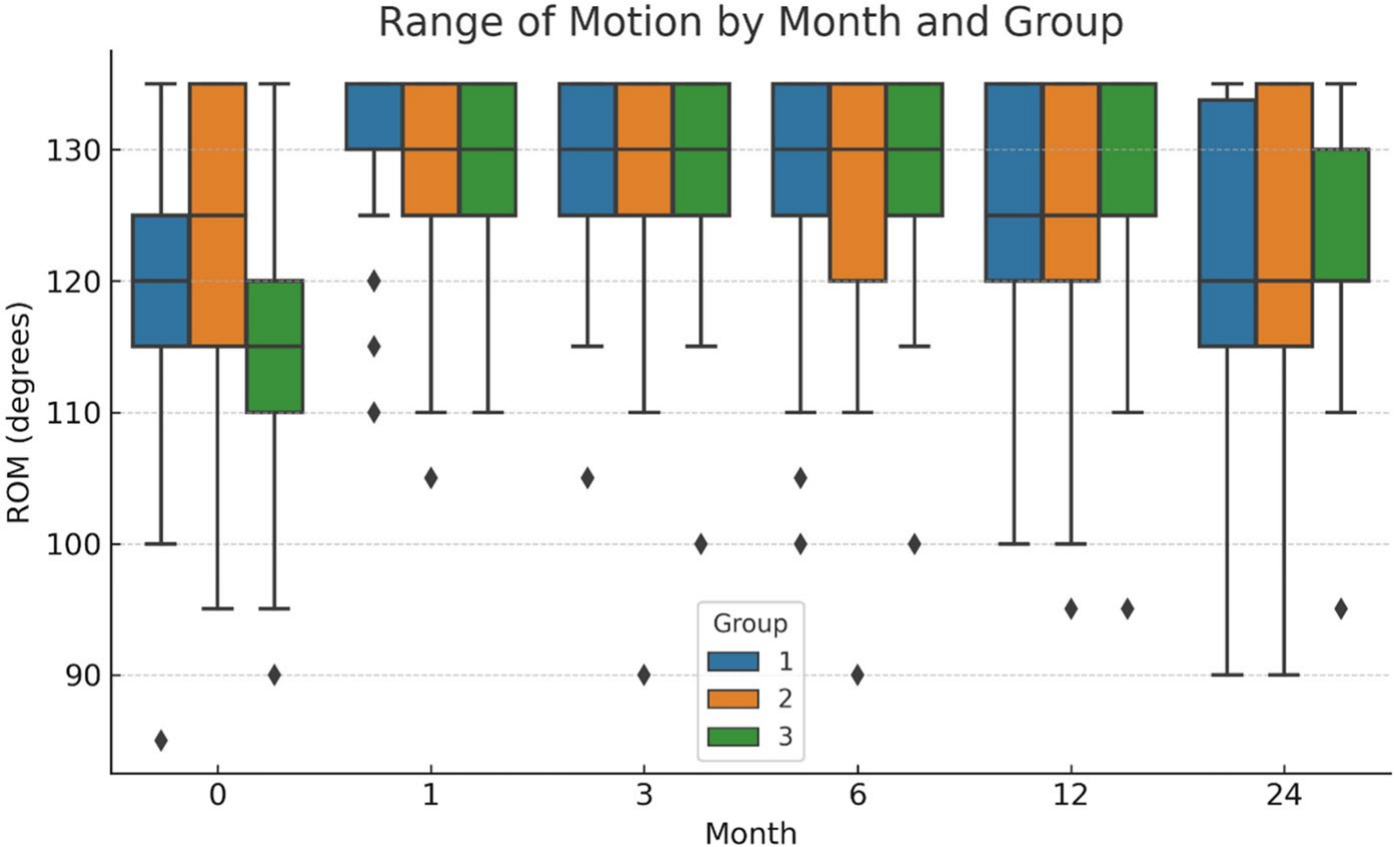
Box plots showing the distribution of the range of motion (ROM) values at baseline and follow‐up months (1, 3, 6, 12, and 24) for each mechanical axis alignment group. Boxes represent the interquartile range (IQR) with medians shown as horizontal lines; whiskers extend to 1.5 × IQR and outliers are plotted as individual points. Significant between‐group differences were observed at baseline (*p* < 0.001) and at 1 month (*p* = 0.024).

Range of motion (ROM) values varied across groups during the 24‐month follow‐up. At baseline, Group 1 had a mean ROM of 123.5° ± 10.9° (median 125 [95–135]), Group 2 had 120.7° ± 9.5° (median 120 [85–135]), and Group 3 had 115.0° ± 10.8° (median 115 [90–135]), with Group 3 demonstrating significantly lower values compared to Groups 1 and 2 (*p* < 0.001). At 1 month, all groups exhibited improvements, with Group 2 reaching the highest mean ROM of 130.5° ± 8.7° (median 131 [110–140]); between‐group differences were statistically significant (*p* = 0.024). No significant differences were observed at 3 or 6 months. At 12 and 24 months, ROM values declined in all groups (Group 1: 121.4° ± 9.2°, Group 2: 119.7° ± 10.1°, Group 3: 113.9° ± 11.3°), with the most pronounced decrease in Group 3, although these differences were not statistically significant (Figure 6).

### 3.6 Regression Analyses

Additional regression analyses were conducted to evaluate the impact of the mechanical axis angle (MAA) on the outcomes of PRP treatment. In univariate models, MAA demonstrated a significant association with the KOOS Total change from baseline to 6 months (*β* = 1.043, *p* = 0.001); however, it did not show a significant relationship with changes in VAS (*p* = 0.128) or KUJALA scores (*p* = 0.519). In multivariate models that adjusted for age and BMI, MAA continued to be significantly associated with KOOS Total change (*β* = 1.017, *p* = 0.002) and approached significance for VAS change (*β* = −0.074, *p* = 0.084). Furthermore, a higher BMI was independently linked to smaller improvements in VAS scores (*β* = −0.139, *p* = 0.018).

## 4 Discussion

### 4.1 Principal Findings

The primary finding of this study indicates that an increased lower extremity mechanical axis diminishes the effectiveness of PRP treatment on pain relief and knee function scores. In all patient groups that received three doses of PRP treatment, pain and clinical scores reached their highest levels at 6 months before showing a tendency to decline. The effectiveness of PRP treatment, assessed by the initial KOOS values, was found to persist for 12 months in all groups, which is consistent with the findings of studies conducted by Kon et al. [20] and Patel et al. [21]. In patients with K/L grade IV OA, similar to the findings of Saraf et al. [22], three doses of intra‐articular PRP injections resulted in slight improvements in short‐term subjective pain and knee function scores. Importantly, these treatments did not lead to any major complications [22]. Moreover, there were no significant differences between the values at 24 months and the initial values across all groups in this study. The effectiveness of PRP treatment was notably lower in patients with severe varus deformities compared to the other groups. Therefore, we do not recommend PRP treatment for patients with severe varus.

### 4.2 Comparison With Previous Studies and Clinical Implications

Numerous studies have investigated the use of PRP [21, 22, 23, 24, 25, 26, 27, 28, 29, 30, 31, 32, 33, 34, 35, 36, 37, 38, 39, 40, 41, 42, 43, 44]. However, very few of these studies are randomized, controlled trials with scientifically validated results that demonstrate the efficacy of PRP [21, 22, 26, 27, 28, 33, 43, 44, 45, 46, 47, 48]. While the literature suggests that PRP may be effective, there is a notable absence of studies with robust scientific evidence. Furthermore, it remains unclear which patient populations will benefit the most from this treatment. Key details such as the platelet count, leukocyte count, and the specific injection site within the knee are often poorly defined in the studies on PRP. Additionally, the impact of injection frequency on treatment effectiveness is not thoroughly understood. Addressing these uncertainties regarding PRP treatment would require randomized, placebo‐controlled studies involving a larger number of subjects. In this study, we investigated how the alignment of the lower extremity mechanical axis affects the efficacy of PRP treatment. To ensure the validity of our findings, we meticulously controlled various external factors and variables that could potentially influence the outcomes of PRP therapy. By isolating the mechanical axis as a key variable, we aimed to provide a clearer understanding of its role in the effectiveness of PRP treatment for patients.

Effect size analysis showed that patients with greater mechanical axis varus (Group 3) experienced smaller improvements, particularly in KOOS total scores, where medium‐to‐large negative effects were observed compared to Groups 1 and 2. VAS improvements were also smaller in Group 3, with a moderate effect size when compared to Group 2. KUJALA scores showed smaller differences, suggesting that patellofemoral function was less affected than overall knee function. These findings reinforce the notion that increased varus alignment reduces the functional benefit of PRP injections.

Regression analyses in the present study demonstrated that greater varus alignment, as measured by the mechanical axis angle (MAA), was an independent predictor of reduced functional improvement following PRP treatment, particularly in KOOS Total scores, even after adjusting for age and BMI. This finding aligns with previous reports indicating that malalignment, especially varus deformity, accelerates medial compartment loading and cartilage degeneration, thereby diminishing the potential benefits of biologic therapies such as PRP [9, 14]. The borderline association between MAA and VAS change observed in our analysis is consistent with studies showing that malalignment primarily impacts functional outcomes rather than pain perception [12]. Furthermore, the independent negative effect of higher BMI on pain improvement corroborates earlier evidence that excess body weight exacerbates joint load and inflammation, reducing the efficacy of intra‐articular therapies [49, 50]. Collectively, these results emphasize the need to consider both mechanical alignment and BMI in treatment planning, echoing recommendations from recent clinical guidelines that patient selection should integrate biomechanical and systemic factors to optimize PRP therapy outcomes in knee osteoarthritis [51].

There is a notable relationship between the alignment of the lower extremity in the frontal plane and the development of OA [9, 10]. Numerous studies have demonstrated the connection between lower extremity alignment disorders and both the onset and progression of OA [9, 10, 11, 12, 13, 14]. Changes in the geometry of the femur, tibia, hip, knee, and ankle joints influence the anatomical tibiofemoral angle (aTFA) used to assess alignment. Consequently, the measurement of the mechanical tibiofemoral angle (mTFA) yields more reliable and accurate results for determining alignment in patients with OA [19]. The alignment of the lower extremity is influenced by the geometric characteristics of the femur, tibia, and joints, necessitating an examination of the entire bone structure of the femur and tibia, as well as the joint architecture. The knee joint should be assessed in full extension while bearing weight on the foot, which allows for visualization of the femoral head and ankle joint [52]. Research has shown that measurements based on the mechanical axis present a smaller margin of error compared to those based on the anatomical axis [52, 53]. In this study, when calculating the deformity angles of the patients, the angle between FMA and TMA, namely mTFA, was taken as the reference deformity angle. Also known as the ‘HKA” angle, it was used.

Since the patella faces forward, deformities related to it are often observed in radiographs taken with this orientation as a reference, which can lead to malrotation [54]. Therefore, we chose not to use the patella as a reference point in the alignment radiographs for this study. The positioning of the lower extremity is crucial for standardizing and ensuring the reproducibility of lower extremity alignment radiographs. Factors such as the rotation of the extremities, the position of the foot, and the flexion of the knee joint influence the medial tibial‐femoral angle (mTFA) [52, 55, 56].

In the current study, we measured the lower extremity mechanical axis angles of 210 patients who underwent PRP treatment. A moderate negative correlation was observed between the Mechanical Axis Angle and the changes in scores on the VAS, KOOS, and KUJALA from the baseline to the 6‐month mark. As the mechanical axis angle increased, we noted detrimental effects on pain levels and clinical scores. The literature includes a clinical study exploring the relationship between PRP treatment and the lower extremity mechanical axis angle [44]. While PRP therapy impacts the biochemical environment within the joint, it does not directly modify the joint's structure. Therefore, we do not recommend PRP treatment for patients with advanced varus deformities; instead, surgical treatment options should be considered for those exhibiting significant structural and anatomical changes in the knee joint.

Various techniques can be employed to prepare PRP, resulting in differing concentrations of platelets in PRP treatments [57]. Research has shown that a concentration of 1,000,000 platelets per microliter significantly enhances bone and soft tissue healing. Accordingly, this specific concentration in a 5‐mL plasma volume is currently recognized as the standard definition of PRP. Lower platelet concentrations are not considered effective for improving wound healing, while higher concentrations have yet to demonstrate any additional advantages [58]. In this study, type 2A PRP was applied to all patients according to the Mishra classification [18].

In the current study, we investigated the effects of three different doses of PRP on pain management and clinical outcomes. Our findings demonstrated that all three doses significantly reduced pain levels and improved clinical scores among participants. This observed effectiveness of multiple doses of PRP supports previous research in the field, which has similarly indicated that a multi‐dose approach can enhance therapeutic results [46, 59, 60].

Patients with greater varus alignment (Group 3) demonstrated a lower range of motion (ROM) at baseline compared to other groups, and although all groups experienced early improvements following PRP treatment, these gains diminished over time. By the 12‐ and 24‐month follow‐up, ROM values had declined in all groups, with the largest decrease observed in Group 3. While these late differences were not statistically significant, the trend suggests that patients with higher varus deformity may have a reduced capacity to maintain long‐term improvements in joint mobility after PRP therapy. This finding highlights the potential importance of considering baseline alignment in both treatment planning and post‐treatment follow‐up strategies.

The thorough evaluation of the patellofemoral joint is critical prior to the administration of platelet‐rich plasma (PRP) injections in patients. This assessment helps to ensure the optimal efficacy of the treatment and to identify any underlying conditions that may affect outcomes. To date, the existing literature includes only two clinical trials that have specifically examined the relationship between PRP therapy and the patellofemoral joint, underscoring a gap in research in this area [44, 61]. Of these studies, only one has utilized the KUJALA scoring system to evaluate the outcomes associated with PRP treatment [44]. In their findings, Jang et al. reported that the presence of patellofemoral joint degeneration negatively influenced the results of PRP treatment, suggesting that degenerative changes in the joint could hinder the effectiveness of the therapy [61]. In contrast, Yurtbay et al. reported evidence that PRP treatment has a beneficial impact on the KUJALA score, which is commonly used to assess knee function and pain levels in patients with patellofemoral disorders [44]. This indicates that PRP may refine patient outcomes and improve functional scores even in cases where degeneration is present. Moreover, a recent study utilizing MRI technology has revealed that PRP treatment significantly increases the cartilage volume within the patellofemoral joint [62]. This data suggests that PRP injections may not only enhance pain relief and functionality but also contribute to the regeneration of cartilage, which is a critical factor for joint health. This new double‐blind randomized clinical trial provides important corroborative evidence that supports the positive relationship between PRP treatment and the KUJALA score, aligning with the results observed in our own study. Together, these findings highlight the need for further investigation into the therapeutic potential of PRP in treating disorders of the patellofemoral joint.

### 4.3 Strengths of the Study

The strengths of this study stem from our diligent efforts to ensure the validity of our findings. We included patients with similar characteristics and factors that could potentially influence the outcomes of PRP treatment, such as age, BMI, and PRP preparation technique. By isolating the mechanical axis as a key variable, we aimed to offer a clearer understanding of its role in the efficacy of PRP treatment for patients.

### 4.4 Limitations and Future Directions

The primary limitations of this study include the exclusive use of direct radiographs for imaging during patient follow‐up. Incorporating magnetic resonance imaging (MRI) could have provided more detailed visualization and mapping of articular cartilage changes, thereby allowing for a more objective assessment of PRP's structural effects. Additionally, no biological or biochemical analyses of synovial fluid or other joint tissues were performed before and after treatment, which could have offered valuable insights into the physiological mechanisms underlying PRP's clinical effects. The relatively small sample size within each subgroup and the single‐center design may limit the generalizability of the findings. Furthermore, the follow‐up period, while extending to 24 months, may still be insufficient to capture longer‐term disease progression and treatment effects. Finally, potential confounding factors such as variations in physical activity levels, concomitant treatments, and adherence to rehabilitation protocols were not systematically controlled, which could have influenced the clinical outcomes.

## 5 Conclusions

The findings of this study suggest that PRP has a beneficial effect on pain management and clinical outcomes in the treatment of knee osteoarthritis. However, greater varus alignment, as indicated by an increased mechanical axis angle (MAA), is associated with reduced functional improvement and, to a lesser extent, diminished pain relief, even after adjusting for age and BMI. Higher BMI also independently predicts smaller pain improvements. While PRP treatment can favorably influence the biochemical environment within the joint, it does not directly alter the structural integrity of the knee. Early gains in range of motion (ROM) are observed across all groups but tend to diminish over time, particularly in patients with marked varus deformity. Patellofemoral function appears less affected by MAA. It is advisable to avoid PRP treatment in patients with significant varus or valgus alignment, and for those with pronounced structural or anatomical changes, surgical treatment options should be considered. These findings underscore the importance of evaluating mechanical alignment and BMI during patient selection and counseling, and suggest that adjunctive biomechanical or corrective strategies may enhance PRP therapy outcomes.

## Author Contributions

All authors had full access to the data in the study and take responsibility for the integrity of the data and the accuracy of the data analysis. Conceptualization: A.Y. and F.S. Methodology: A.Y. and F.S. Investigation, A.Y. Formal analysis: A.Y., F.E., and F.S. Resources: A.Y. and F.S. Writing – original draft: A.Y. and F.S. Writing – review and editing: A.Y. and F.S. Visualization: A.Y. and F.S. Supervision: F.S.

## Disclosure

The authors have nothing to report.

## Ethics Statement

This retrospective study was conducted with the approval of the Samsun University clinical research ethics committee (Approval number: 2023/14/16 Date: 09.08.2023). Institutional Review Board approval and informed consent of the patients were obtained.

## Conflicts of Interest

The authors declare no conflicts of interest.

## Data Availability

The data that support the findings of this study are available on request from the corresponding author. The data are not publicly available due to privacy or ethical restrictions.
